# Combined neutrophil-to-lymphocyte ratio and nomogram for predicting progression-free survival in recurrent/metastatic cervical cancer treated with immune checkpoint inhibitors

**DOI:** 10.3389/fimmu.2026.1811428

**Published:** 2026-04-14

**Authors:** Zhen Xu, Li Zhang, Hongyan Wang, Xia Li, Wenjun Qian, Zhiyan Zhao, Kaiyue Yang, Guiqin Bai

**Affiliations:** 1 Gene Joint Laboratory, The First Affiliated Hospital of Xi’an Jiaotong University, Xi’an, Shaanxi, China; 2 Department of Gynecology and Obstetrics, The First Affiliated Hospital of Xi’an Jiaotong University, Xi’an, Shaanxi, China

**Keywords:** Cervical cancer, immune checkpoint inhibitors, neutrophil-to-lymphocyte ratio, nomogram, prognosis

## Abstract

**Background:**

The outlook for individuals dealing with metastatic or recurrent cervical cancer is still unfavorable, and existing biomarkers that assess the effectiveness of immune checkpoint inhibitors (ICIs) have certain drawbacks. Our objective was to assess the predictive importance of the combined neutrophil-to-lymphocyte ratio (Combined.NLR), incorporating both pre-treatment and post-treatment measurements in this patient population.

**Methods:**

This retrospective cohort analysis, performed at one center, encompassed 148 individuals diagnosed with metastatic or recurrent cervical cancer who underwent treatment with ICIs. Patients were categorized into the Combined.NLR groups according to the median neutrophil-to-lymphocyte ratio (NLR) values obtained before and after treatment. The relationship between the Combined.NLR and progression-free survival (PFS) was examined through Cox regression analysis. A prognostic nomogram that includes various factors was created.

**Results:**

The Combined.NLR was identified as an independent prognostic factor for PFS. Individuals classified in the poor group exhibited a notably increased risk of disease progression in comparison to those in the good group [hazard ratio (HR) = 1.355, 95% confidence interval (CI): 1.006–1.824, p = 0.046]. The nomogram, which integrated the Combined.NLR, histological type, PD-L1 expression, the count of previous treatment lines, and the presence of multi-organ metastasis demonstrated a concordance index of 0.70. Calibration curves showed a strong correlation between the predicted results and the actual outcomes.

**Conclusion:**

The Combined.NLR is a dynamic and readily accessible prognostic marker. The developed nomogram provides individualized risk prediction for individuals with metastatic or recurrent cervical cancer, potentially aiding clinical decision-making.

## 1 Introduction

Cervical cancer remains one of the most common types of cancer impacting women worldwide, posing a considerable public health issue due to its high incidence and mortality rates (1, 2). In 2022, the global reports indicated roughly 662,301 new cases and 348,874 deaths (3). While the prognosis of early-stage cervical cancer has markedly improved through multimodal treatment approaches including surgery, radiotherapy, and concurrent chemoradiotherapy (4, 5), therapeutic options remain severely limited for individuals with metastatic or recurrent disease. Existing treatments often fail to achieve long-term disease control, resulting in generally poor outcomes (6). These results highlight the pressing necessity for innovative and enhanced treatment approaches aimed at increasing survival rates among patients with metastatic or recurrent cervical cancer.

For patients suffering from metastatic or recurrent cervical cancer, the conventional options for systemic treatment primarily include chemotherapy involving platinum-based drugs and targeted therapies such as bevacizumab (7, 8). However, the objective response rates to these therapies are often limited, and they are frequently associated with adverse effects, including myelosuppression and nephrotoxicity. Consequently, achieving long-term survival benefits remains a significant clinical challenge (9, 10). For example, among patients diagnosed with recurrent cervical cancer who are undergoing treatment with single-agent chemotherapy, the median overall survival (OS) is merely 8.5 months (11). Recent studies have demonstrated that immune checkpoint inhibitors (ICIs) exhibit remarkable clinical efficacy across various solid tumors. These agents effectively restore antitumor immune responses through the inhibition of the PD-1/PD-L1 pathway (12, 13). In clinical studies involving metastatic or recurrent cervical cancer, ICIs have shown promising antitumor efficacy, offering a novel therapeutic avenue and renewed hope for this patient population (11, 14).

The use of ICIs in clinical settings has generated fresh optimism for patients; nonetheless, the effectiveness of these therapies differs markedly from one individual to another. Not all patients benefit from ICI treatment, with a considerable proportion exhibiting no response or developing primary resistance (15, 16). Currently used biomarkers for predicting the effectiveness of immune checkpoint inhibitors, such as tumor mutational burden (TMB), PD-L1 expression, and microsatellite instability (MSI), exhibit limitations in their accuracy. For example, PD-L1 level shows intratumoral heterogeneity and lacks a standardized detection threshold; TMB assessment is often costly and time-consuming; and MSI positivity is relatively low in cervical cancer. Moreover, the detection of these biomarkers typically relies on invasive tissue biopsies, which are costly, may have limited accessibility, and are not well-suited for dynamic monitoring (17, 18).

Thus, a distinct clinical requirement exists for the development of innovative biomarkers that are readily obtainable, cost-effective, and capable of reflecting the dynamic tumor microenvironment to optimize patient selection and guide ICI treatment strategies. The neutrophil-to-lymphocyte ratio (NLR) is a metric related to inflammation that is obtained from standard blood tests. Its simplicity and affordability have linked it to outcomes in prognosis and immunotherapy across multiple cancers (19, 20). Significantly, the evolving alterations in NLR and its prognostic significance for individuals with metastatic or recurrent cervical cancer undergoing treatment with ICIs are still not sufficiently understood.

The objective of this research was to assess a novel prognostic indicator for progression-free survival in patients with metastatic or recurrent cervical cancer undergoing immune checkpoint inhibitor treatment by evaluating a Combined.NLR that incorporates both pre- and post-treatment measurements. We additionally aimed to create a functional nomogram that includes this metric to assist in risk stratification and support clinical decision-making.

## 2 Patients and methods

### 2.1 Study design and patient selection

This study, which took a retrospective cohort approach and was performed at a single institution, aimed to evaluate prognostic indicators in patients with recurrent or metastatic cervical cancer following treatment with ICIs. The subjects of this research were patients who received ICI therapy at the First Affiliated Hospital of Xi’an Jiaotong University between January 2022 and December 2024.

The criteria for inclusion were outlined as follows: 1) a cervical cancer diagnosis confirmed through histopathological examination, 2) the disease has advanced to either a metastatic or recurrent stage, 3) at least one dose of a PD-1/PD-L1 inhibitor has been administered, 4) availability of complete clinicopathological records and follow-up data, and 5) complete blood count results should be available from within 7 days before the initial administration of ICIs and at the conclusion of the first treatment cycle, which is roughly 3 to 4 weeks later.

Patients were excluded according to these criteria: 1) diagnosis with another concurrent malignancy, 2) history of severe autoimmune disease or chronic inflammatory disease, 3) lack of essential clinical or laboratory data, and 4) pregnancy or lactation.

Authorization for this research was obtained from the Ethics Committee at the First Affiliated Hospital of Xi’an Jiaotong University, and it was conducted in accordance with the principles outlined in the Declaration of Helsinki. Due to the retrospective nature of the study, the necessity for informed consent was waived.

### 2.2 Data collection

A retrospective analysis of the electronic medical record system was conducted to extract clinical and pathological features. The gathered information encompassed the following: age, histological type, PD-L1 level [evaluated using the Combined Positive Score (CPS)], recurrence status, the number of prior lines of therapy, and the presence of multi-organ metastasis (defined as involvement of two or more distant organs). Additionally, results from complete blood count tests, including neutrophil and lymphocyte counts, were collected both before and after treatment initiation.

The pathological slides used in this study were obtained from the First Affiliated Hospital. All patients were treated at this institution, and the slides were retrospectively collected samples representing different histological subtypes of cervical cancer, including cases with varying levels of PD-L1 expression. The use of these specimens was approved by the institutional ethics committee and complied with all relevant ethical regulations. PD-L1 expression was assessed via immunohistochemistry (IHC) using a standardized staining platform (BenchMark ULTRA PLUS, Roche Diagnostics Co., Ltd., Suzhou, Jiangsu Province) and the Dako PD-L1 antibody (clone 22C3). PD-L1 expression was evaluated using the CPS. All slides were independently assessed by trained pathologists with expertise in PD-L1 evaluation, and all cases underwent blinded dual review. Any discrepancies between the two pathologists were resolved through discussion to reach a consensus.

The primary endpoint of the research was progression-free survival (PFS), defined as the time from the initiation of immune checkpoint inhibitor therapy to either disease progression (according to Response Evaluation Criteria in Solid Tumors (RECIST) 1.1 criteria) or death from any cause. Patient follow-up was conducted until December 2024.

### 2.3 Definition and calculation of the combined NLR metric

The calculation of the NLR was performed by taking the absolute neutrophil count and dividing it by the absolute lymphocyte count, which was acquired from peripheral blood tests. For this research, the median NLR value obtained prior to treatment and the median NLR value recorded following treatment served as the respective cutoff thresholds. Patients were categorized into three groups to establish a Combined.NLR variable based on the pre- and post-treatment NLR levels in relation to these medians:

Good group: Patients with both pre-treatment NLR and post-treatment NLR below their respective median values.

Poor group: Patients with both pre-treatment NLR and post-treatment NLR above their respective median values.

Intermediate group: Patients who did not fulfill the criteria for the good or poor categories were defined by having pre-treatment NLR or post-treatment NLR exceeding the median value.

### 2.4 Statistical analysis

Statistical evaluations were performed using R version 4.4.2. Continuous variables were expressed as medians with their corresponding interquartile ranges (IQRs), while categorical variables were presented as counts and percentages. Depending on the distribution of the data, the Mann–Whitney U test or the Kruskal–Wallis H test was applied to assess differences among groups for continuous data. Categorical variables were analyzed using either the chi-square test or Fisher’s exact test.

The Kaplan–Meier technique was employed to evaluate PFS, and the survival curves were examined using the log-rank test. In order to determine independent prognostic factors for PFS, both univariate and multivariate Cox proportional hazards regression models were applied. Results were reported as hazard ratios (HRs) accompanied by their respective 95% confidence intervals (CIs). The variables selected for the multivariate analysis were based on their significance in the univariate analysis or their established clinical relevance.

A nomogram aimed at predicting PFS was created based on the independent prognostic variables identified in the multivariate Cox regression analysis. The performance of the nomogram was evaluated by assessing its discriminative ability using the concordance index (C-index) and analyzing its calibration through calibration curves. Internal validation was conducted using the bootstrap resampling method with 1,000 repetitions. Statistical analyses were executed with two-sided significance, defining a p-value of less than 0.05 as statistically significant.

## 3 Results

### 3.1 Patient baseline characteristics

**Table 1** summarizes the clinicopathological features of the patients included in the study. The median age of the participants was 49 years, with a range from 28 to 74 years. Squamous cell carcinoma was the predominant histological type (112 patients, 75.68%). PD−L1 expression status, assessed using the CPS, was <1 in 11 patients (7.43%), 1–20 in 71 patients (47.97%), and >20 in 66 patients (44.59%). Regarding prior treatment lines, 59 patients (39.86%) had received one line, 53 patients (35.81%) two lines, and 36 patients (24.32%) three or more lines. Sixty−nine patients (46.62%) had recurrent disease, and 29 patients (19.59%) presented with multi−organ metastasis.

**Table 1:** Baseline clinicopathological characteristics of patients (N = 148).

Characteristic	Value/no. of patients (%)
Demographics	
Age (years)	49 (28–74)
Histological type	
Squamous cell carcinoma	112 (75.68%)
Others	36 (24.32%)
PD-L1 expression status (CPS)	
<1	11 (7.43%)
1–20	71 (47.97%)
>20	66 (44.59%)
Prior treatment lines	
1	59 (39.86%)
2	53 (35.81%)
≥3	36 (24.32%)
Recurrent tumor	
Yes	69 (46.62%)
No	79 (53.38%)
Multiple-organ metastasis	
Yes	29 (19.59%)
No	119 (80.41%)

CPS, Combined Positive Score.

The median pre-treatment NLR was 2.401 (range, 0.528–8.985), and the median post−treatment NLR was 2.231 (range, 0.556–8.420). According to the Combined.NLR definition, 38 patients (25.68%) were classified into the good group, 38 (25.68%) into the poor group, and 72 (48.65%) into the intermediate group.

### 3.2 Association between Combined.NLR and PFS

**Figure 1:**
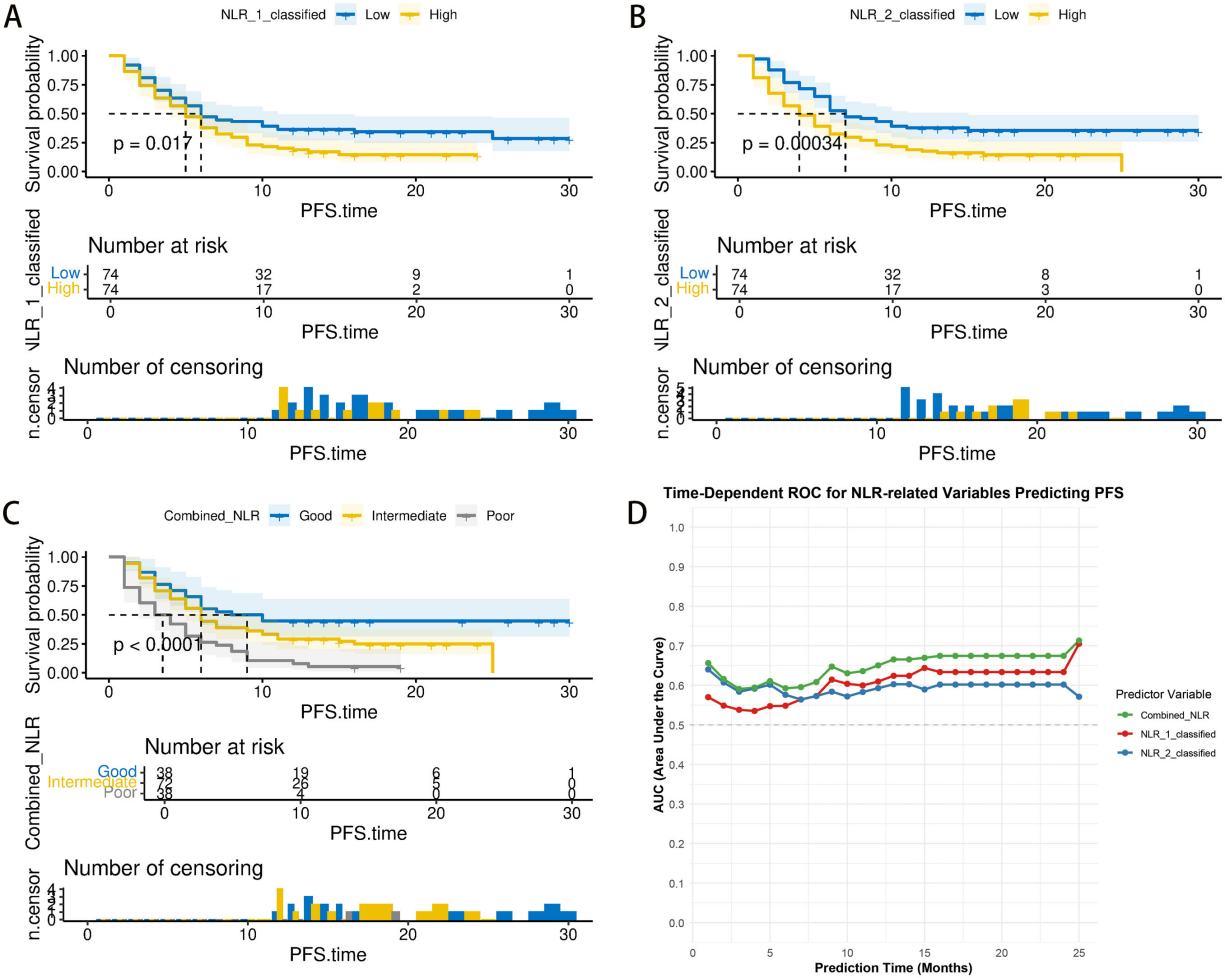
Comparison of progression-free survival based on combined and single-time-point neutrophil-to-lymphocyte ratio (NLR) measurements in patients with metastatic or recurrent cervical cancer treated with immune checkpoint inhibitors (ICIs). **(A)** The Kaplan–Meier curves illustrate progression-free survival (PFS) for patients categorized solely based on their pre-treatment NLR. A notable difference in PFS was noted among the groups (log-rank p = 0.017). **(B)** Kaplan–Meier curves depicting PFS for patients stratified by the post-treatment NLR alone. A significant difference in PFS was also observed between these groups (log-rank p < 0.001). **(C)** Kaplan–Meier curves depicting progression-free survival (PFS) for patients stratified by the combined neutrophil-to-lymphocyte ratio (Combined.NLR) (derived from both pre- and post-treatment values). Patients with a high Combined.NLR experienced significantly worse PFS (log-rank p < 0.001). **(D)** Time-dependent receiver operating characteristic (ROC) curve analysis comparing the predictive accuracy of the Combined.NLR, pre-treatment NLR, and post-treatment NLR for PFS. The Combined.NLR demonstrated superior predictive capability compared to using NLR measurements from a single time point.

The Kaplan–Meier survival analysis demonstrated notable variations in PFS for patients categorized by the Combined.NLR (log-rank p < 0.001). While differences in PFS were noted for groups categorized by pre-treatment NLR alone (log-rank p = 0.017) and post-treatment NLR alone (log-rank p < 0.001; **Figures 1A–C**), the time-dependent receiver operating characteristic (ROC) curve analysis demonstrated that the Combined.NLR exhibited superior predictive power for PFS in patients suffering from metastatic or recurrent cervical cancer undergoing ICI treatment, in contrast to depending exclusively on individual NLR measurements at specific time points (**Figure 1D**).

To further validate the robustness of the median-based cutoff strategy, we additionally performed time-dependent ROC curve analysis to determine the optimal thresholds for NLR_1, NLR_2, and the Combined.NLR at the 6-month time point. The ROC-derived cutoff values and their corresponding Kaplan–Meier survival analyses are presented in **Supplementary Figure 1**. Although the time-dependent ROC approach yielded slightly improved predictive performance, as reflected by modest increases in Area Under the Curve (AUC) values, the overall survival discrimination remained consistent with the median-based grouping.

Considering the potential risk of overfitting associated with data-driven cutoff optimization, we retained the median values as the primary grouping method to enhance the generalizability and clinical applicability of the Combined.NLR.

To evaluate the methodological validity of constructing the Combined.NLR based on the median values of NLR_1 and NLR_2, restricted cubic spline (RCS) analyses were performed. As shown in **Supplementary Figure 2**, both NLR_1 and NLR_2 exhibited a predominantly linear association with progression-free survival, with no significant evidence of non-linearity. These findings support the appropriateness of the median-based Combined.NLR strategy.

**Figure 2:**
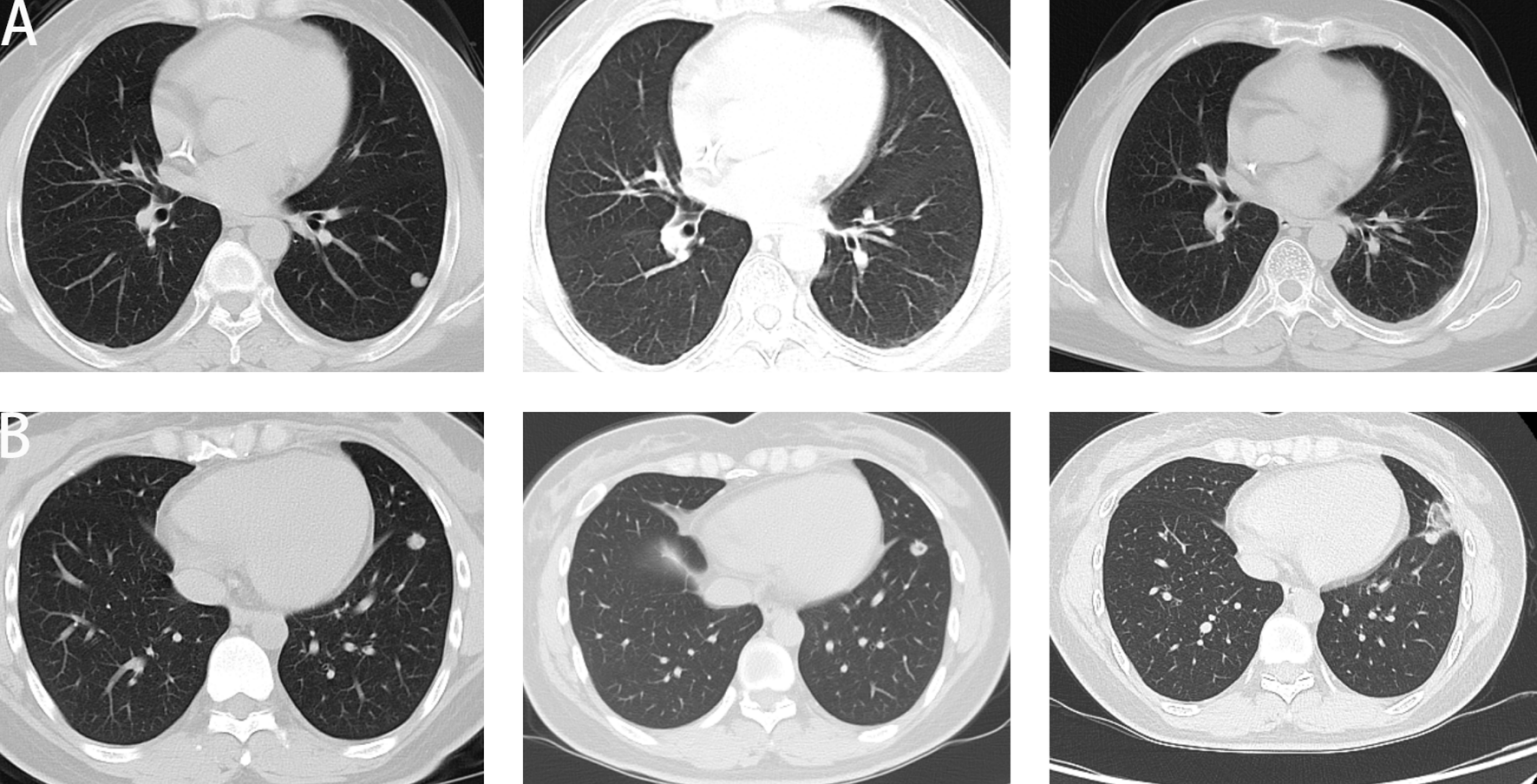
Representative CT images of lung metastases in patients with different responses to immune checkpoint inhibitors (ICIs). **(A)** CT image from a patient with a sustained response showing a reduction in pulmonary metastatic lesions following ICI treatment. **(B)** CT image from a patient with disease progression illustrating the continuous enlargement of metastatic lesions during ICI therapy.

**Figure 2A** shows a lung CT of a patient with a sustained response, where a reduction in metastases can be observed. **Figure 2B** shows a lung CT of a patient with disease progression, where a continuous increase in metastases can be seen.

**Figure 3:**
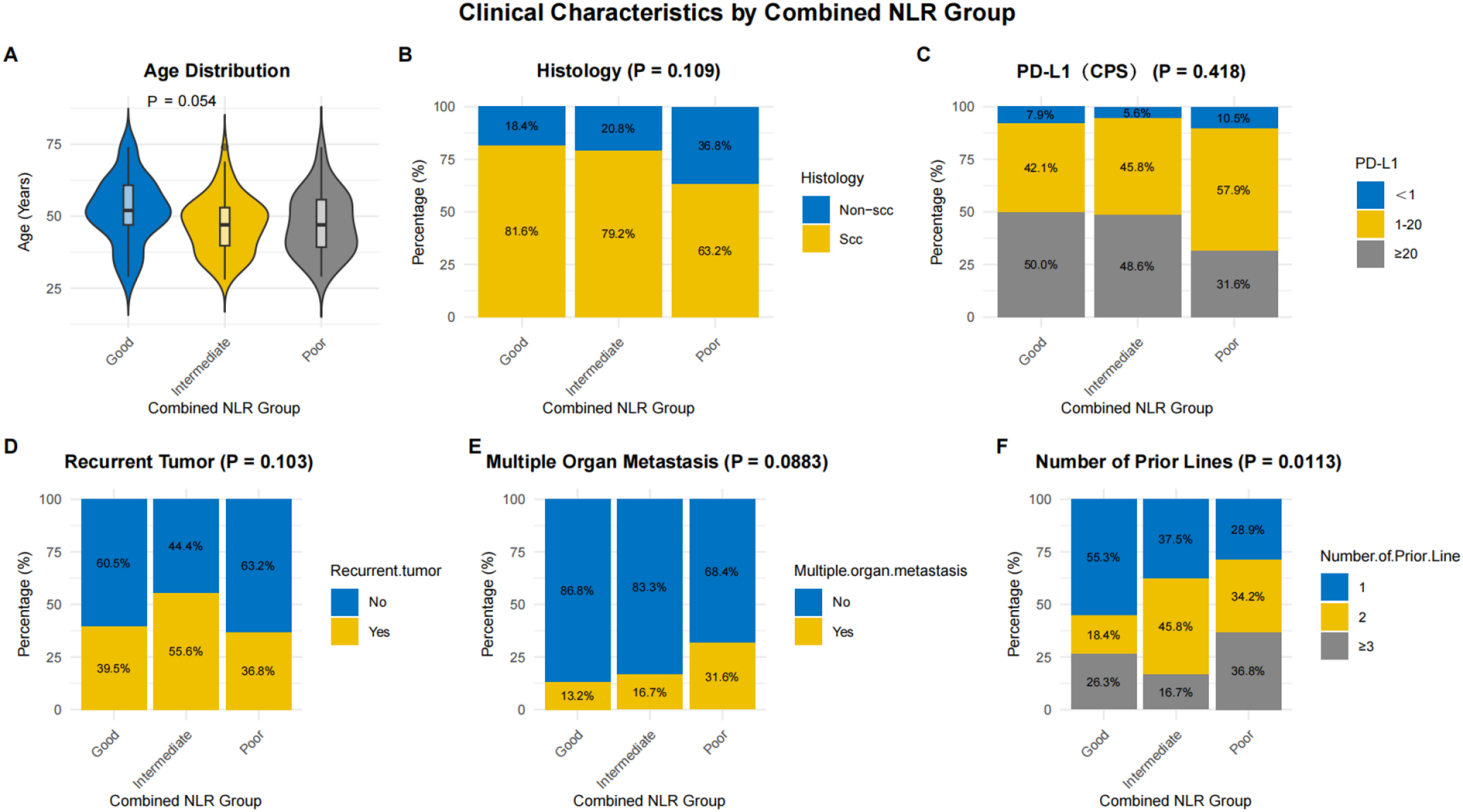
Baseline clinicopathological characteristics of patients stratified by combined neutrophil-to-lymphocyte ratio (Combined.NLR) groups. **(A–F)** Stacked bar charts illustrating the distribution of key clinicopathological variables between the good, intermediate, and poor Combined.NLR groups.

**Figure 4:**
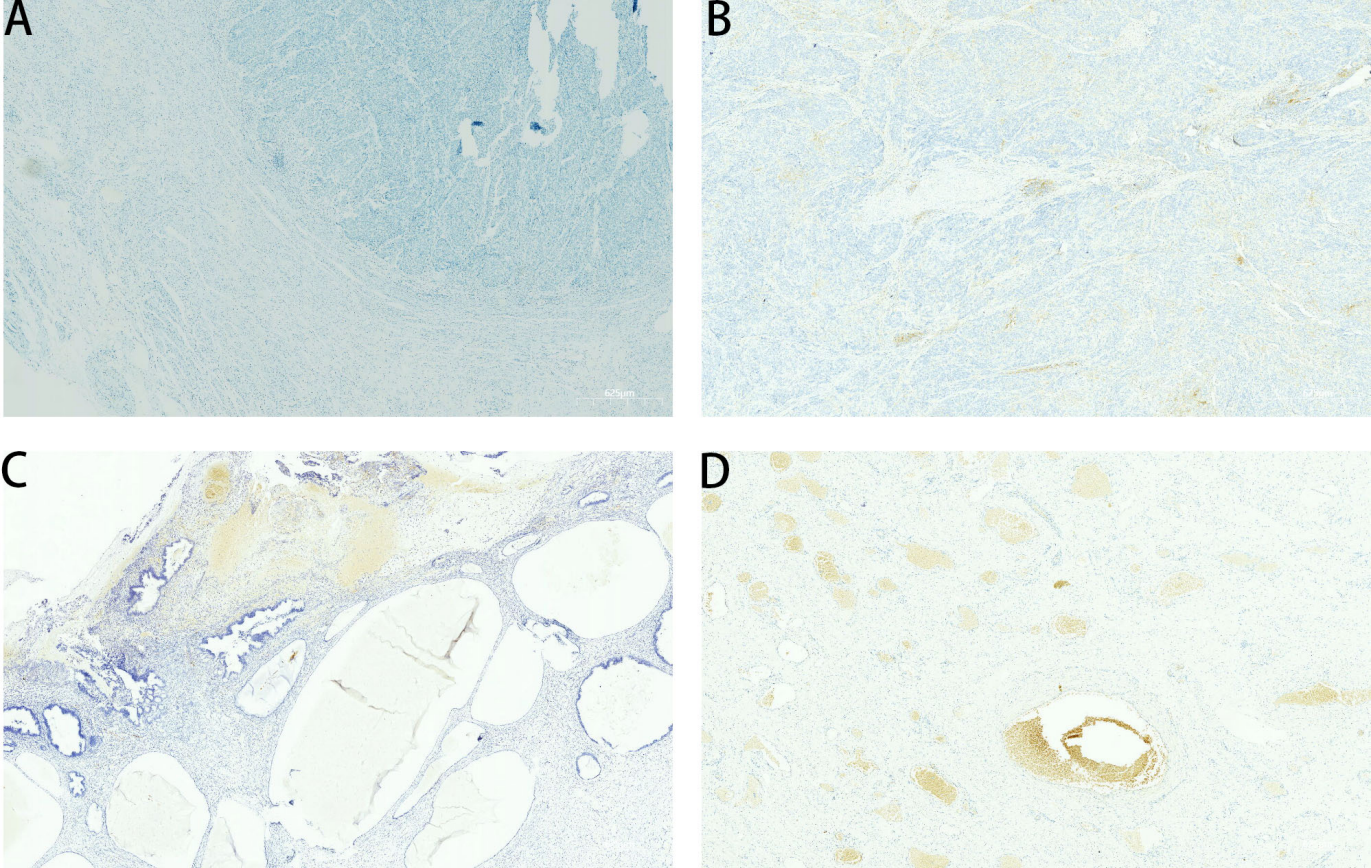
Immunohistochemistry analysis of PD-L1 level [Combined Positive Score (CPS)] in tumor samples. **(A–D)** Representative immunohistochemistry images showing PD-L1 expression categorized by CPS scores. **(A)** CPS < 1. **(B)** CPS 1–5. **(C)** CPS 5–20. **(D)** CPS > 20.

A review of the initial characteristics within the Combined.NLR groups revealed a statistically significant difference in the number of previous lines of therapy (p = 0.0113). Patients classified in the poor group typically underwent a greater number of prior therapy lines than those categorized in the good group. No notable differences were noted between the groups concerning other clinicopathological variables, such as age, histological classification, PD−L1 (CPS), recurrent tumors, and the occurrence of multi-organ metastasis (**Figures 3A–F**). **Figures 4A–D** respectively show the immunohistochemistry results for CPS < 1, CPS 1–5, CPS 5–20, and CPS > 20.

**Figure 5:**
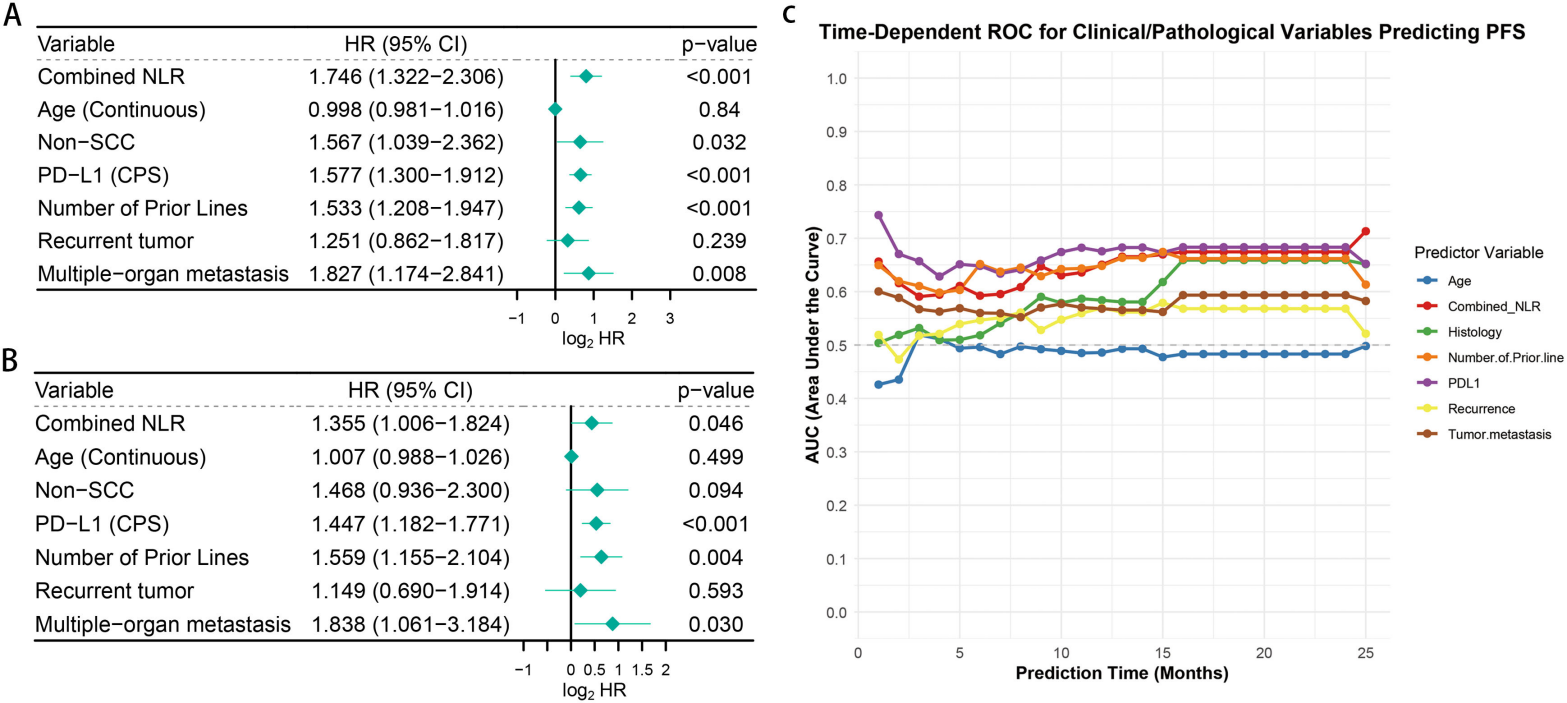
Univariate and multivariate analyses of factors associated with progression-free survival. **(A)** Forest plot showing the univariate Cox proportional hazards regression analysis of clinicopathological factors and patient progression-free survival (PFS). **(B)** Forest plot showing the multivariate Cox regression analysis. **(C)** Time-dependent receiver operating characteristic (ROC) curve analysis comparing the predictive performance of combined neutrophil-to-lymphocyte ratio (Combined.NLR) and clinicopathological characteristics for PFS.

The analysis using univariate Cox proportional hazards regression revealed various factors linked to PFS. These included the Combined.NLR (HR = 1.746, 95% CI: 1.322–2.306, p < 0.001), histological type (squamous cell carcinoma versus other types: HR = 1.567, 95% CI: 1.039–2.362, p = 0.032), PD-L1 expression (HR = 1.577, 95% CI: 1.300–1.912, p < 0.001), the number of prior treatment lines (HR = 1.533, 95% CI: 1.208–1.947, p < 0.001), and the existence of multi-organ metastasis (HR = 1.827, 95% CI: 1.174–2.841, p = 0.008) (**Figure 5A**).

The multivariate analysis illustrated that several independent prognostic factors for PFS in patients with metastatic or recurrent cervical cancer undergoing ICI therapy included the Combined.NLR (HR = 1.355, 95% CI: 1.006–1.824, p = 0.046), PD-L1 expression (HR = 1.447, 95% CI: 1.182–1.771, p < 0.001), the count of previous therapy lines (HR = 1.559, 95% CI: 1.155–2.104, p = 0.004), and the existence of multi-organ metastasis (HR = 1.838, 95% CI: 1.061–3.184, p = 0.030) (**Figure 5B**).

The analysis of time-dependent ROC curves also showed that the predictive capability of the Combined.NLR for PFS was similar to that of recognized clinical attributes like PD-L1 (CPS) status (**Figure 5C**).

### 3.3 Construction and validation of the prognostic nomogram

A nomogram was created to predict PFS in individuals with metastatic or recurrent cervical cancer receiving immunotherapy, utilizing the independent prognostic factors identified through multivariate Cox regression analysis. This nomogram integrates the Combined.NLR, histological type, PD-L1 (CPS) status, the number of prior lines of therapy, and the presence of multi-organ metastasis.

**Figure 6:**
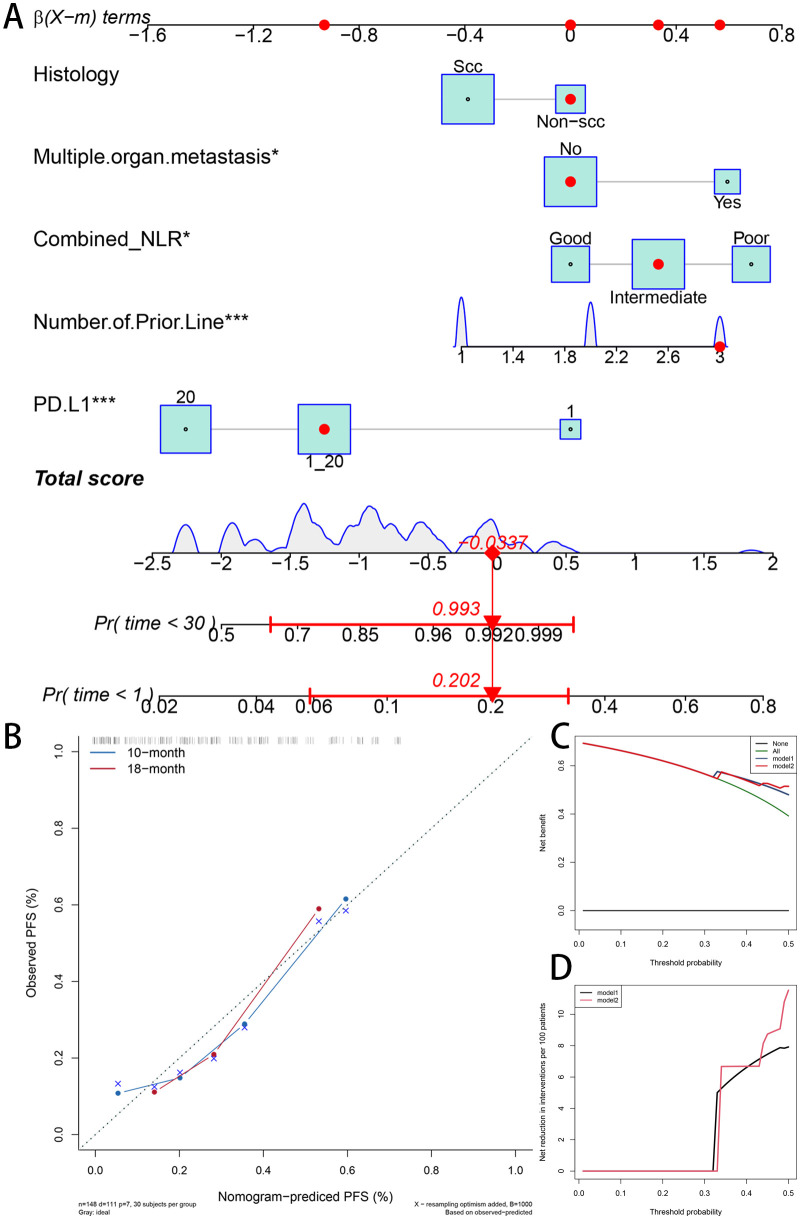
Nomogram for predicting progression-free survival in patients with metastatic or recurrent cervical cancer receiving immune checkpoint inhibitor (ICI) therapy. **(A)** Nomogram integrating five independent prognostic factors identified by multivariate analysis: combined neutrophil-to-lymphocyte ratio (Combined.NLR), histological type, PD-L1 [Combined Positive Score (CPS)] status, number of prior lines of therapy, and presence of multi-organ metastasis. Points are assigned to each variable based on its contribution to progression-free survival (PFS). **(B)** Calibration curves for the nomogram predicting 10-month and 18-month PFS. The curves demonstrate good agreement between nomogram-predicted probabilities and actual observed PFS outcomes. **(C, D)** Decision curve analysis (DCA) evaluating the clinical utility of the nomogram. The y-axis represents net benefit, and the x-axis represents threshold probabilities. The nomogram including Combined.NLR exhibited superior net benefit and net reduction across a range of threshold probabilities compared to the model without Combined.NLR and the “treat-all” or “treat-none” strategies.

The internal validation indicated that the nomogram demonstrated robust discriminative ability, reaching a C-index of 0.70. Calibration curves revealed a favorable correlation between the predicted and actual probabilities for 10-month and 18-month PFS. Additionally, the decision curve analysis (DCA) demonstrated that incorporating the Combined.NLR into the model led to a significant clinical benefit across different threshold probabilities (**Figures 6A–D**).

## 4 Discussion

This study’s findings indicate that the Combined.NLR, a measurement categorized by both pre- and post-treatment NLR levels, acts as a standalone prognostic indicator for PFS in patients with metastatic or recurrent cervical cancer who are receiving ICI therapy. Furthermore, a prognostic nomogram incorporating the Combined.NLR, histological type, PD-L1 (CPS) status, the number of prior therapy lines, and the presence of multi-organ metastasis was developed, which provides a tool for predicting therapeutic efficacy in patients who underwent ICI therapy. These results provide an innovative tool for assessing risk in clinical settings and could assist in developing personalized treatment options for this group of patients.

As an easily accessible inflammatory marker, the NLR reflects the balance of the host immune status (21). An elevated NLR typically indicates increased neutrophil counts and decreased lymphocyte counts, which are associated with an immunosuppressive state within the tumor microenvironment (20, 22). Neutrophils can enhance tumor development, promote angiogenesis, and facilitate metastasis by secreting cytokines, chemokines, and growth factors. In contrast, it is lymphocytes—particularly T cells—that play a crucial role in orchestrating antitumor immune responses (23–25). The research revealed that the predictive accuracy of the Combined.NLR exceeded that of the NLR evaluated at a singular time point. This superiority may be linked to the Combined.NLR’s enhanced capability to thoroughly reflect the fluctuations in a patient’s immune condition. The pre-treatment NLR reflects the baseline inflammatory and immune state prior to ICI therapy, while the post-treatment NLR may indicate the early response to immune intervention or adaptive immune changes during disease progression. By integrating NLR information from both time points, the Combined.NLR reduces the potential variability associated with a single measurement, thereby providing more stable and accurate prognostic information, which is consistent with the time-dependent ROC curve analysis. Accordingly, it can be speculated that the good, intermediate, and poor Combined.NLR subgroups may correspond to varying degrees of immunosuppression or immunogenic potential during ICI treatment, ultimately influencing patient response. This study’s recognition of the Combined.NLR as a predictive indicator for ICI effectiveness is consistent with the prognostic significance that NLR has shown in immunotherapy for various other cancers, including melanoma, lung cancer, and gastric cancer (26, 27). In contrast to biomarkers like the PD-L1 expression and TMB, the Combined.NLR is obtained from standard blood tests, providing benefits such as easy access, affordability, and robust reproducibility. These characteristics enhance its potential for broader application in clinical practice.

Prior research on the prognostic value of NLR in cervical cancer has predominantly focused on single-time-point measurements. For instance, Chen et al. demonstrated in a cohort of 105 patients that an elevated NLR was associated with shorter PFS in cervical cancer patients receiving ICIs (28). Similarly, a retrospective study of 49 patients by Calo et al. suggested that pre-treatment NLR may hold prognostic value for metastatic/recurrent cervical cancer patients treated with PD-1/PD-L1 inhibitors (29). While Du et al. evaluated the relationship between pre-operative, post-operative NLR, and NLR changes in 203 stage I–IIA cervical cancer patients, their study, which categorized patients based on NLR increase or decrease post-treatment, did not observe significant prognostic differences between these groups (30). In contrast to these prior investigations, our study pioneers the exploration of the prognostic utility of the Combined.NLR—integrating both pre- and post-treatment NLR levels—specifically in recurrent or metastatic cervical cancer patients undergoing ICI therapy. Unlike classifications based solely on NLR elevation or reduction, the Combined.NLR effectively mitigates the limitations of single-measurement predictive failures by incorporating dynamic changes, thereby enhancing predictive stability and demonstrating superior predictive capabilities compared to single-time-point NLR measurements. This innovative approach suggests that the Combined.NLR holds significant potential as a robust prognostic biomarker.

In addition to the Combined.NLR, this study’s multivariate analysis recognized histological type, PD-L1 (CPS) status, the number of previous treatment lines, and the existence of multi-organ metastasis as independent prognostic indicators for PFS in patients undergoing ICI therapy for metastatic or recurrent cervical cancer. Higher PD-L1 expression correlates with increased response rates to ICI treatment (14, 31, 32). In contrast, the reaction to ICIs may vary between squamous cell carcinoma and cervical adenocarcinoma, with the latter frequently exhibiting unique biological behaviors and characteristics of the tumor microenvironment (33, 34). Furthermore, an increased number of prior therapy lines and the presence of multi-organ metastasis typically indicate a greater disease burden and more advanced stage, which are correlated with poorer patient prognosis (35, 36). Of note, an association was observed between the Combined.NLR groups and the number of prior lines of therapy in this study. This suggests that the Combined.NLR may not only reflect a patient’s immune status but could also be correlated with the extent of disease progression or treatment history. This result provides additional evidence for the idea that administering PD-1/PD-L1 inhibitors earlier may more effectively promote a positive immune environment, which could potentially improve the response to treatment (37).

The nomogram developed in this study, which integrates the Combined.NLR with multiple clinicopathological features, provides a predictive model for PFS in patients with metastatic or recurrent cervical cancer undergoing ICI therapy. As a visual predictive tool, its intuitive design and ease of use offer clear potential for clinical application (36, 38). By leveraging this model, clinicians can accurately estimate individual patient risk, thereby facilitating the identification of those most likely to derive significant benefit from ICI treatment. Conversely, it also helps pinpoint patients who may necessitate closer monitoring or the exploration of alternative therapeutic strategies due to higher risk. This multi-factor predictive model, in contrast to single biomarkers, enables a more comprehensive and nuanced risk assessment, ultimately supporting optimized, individualized treatment decisions and potentially leading to improved patient outcomes. For instance, patients categorized with a “poor” Combined.NLR status may particularly benefit from more intensive surveillance, such as increased frequency of routine CT scans, or the consideration of advanced imaging modalities like PET-CT, to enable earlier detection of progression or recurrence and guide timely intervention.

While this study offers valuable preliminary insights, several limitations warrant consideration. First, its inherent retrospective and single-center design, coupled with a modest sample size of 148 participants, inevitably restricts the generalizability and external validity of our findings. This design is susceptible to selection bias and may lack sufficient statistical power for robust subgroup analyses, underscoring the need for larger, prospective, multicenter studies to validate these results across diverse populations. Second, despite efforts to optimize, the determination of the Combined.NLR threshold using median values, rather than more statistically sophisticated methods like time-dependent ROC analysis, may not fully maximize prognostic discrimination, although we prioritize its broader applicability. Third, our study acknowledges the absence of independent external validation for the developed nomogram and the lack of comprehensive molecular features (e.g., Human Papillomavirus (HPV) status, TMB, and MSI), which are crucial for a more complete understanding of ICI response and the nomogram’s predictive robustness. Future research should address these gaps by incorporating external cohorts and comprehensive molecular profiling. Additionally, the cutoff values and grouping criteria for the Combined.NLR were derived from the median values within this specific cohort. Future studies are needed to refine the optimal clinical cutoff points, possibly using larger datasets or more sophisticated statistical methods.

Considering these limitations, upcoming studies ought to include prospective, multicenter clinical trials to enhance the validation of the Combined.NLR’s predictive accuracy and clinical usefulness, along with the nomogram that has been created. It would also be valuable to explore the integration of the Combined.NLR with other novel biomarkers—such as genomic, proteomic, or metabolic profiles—to potentially enhance the precision of ICI efficacy prediction. Ultimately, exploring the fundamental cellular and molecular processes through which the Combined.NLR impacts the response to ICIs may clarify its biological foundation and guide the creation of more effective immunotherapy approaches for cervical cancer.

## 5 Conclusion

The findings of this study indicate that the Combined.NLR, classified based on the neutrophil-to-lymphocyte ratio measurements taken prior to and following treatment, serves as an independent prognostic marker for progression-free survival in individuals suffering from metastatic or recurrent cervical cancer receiving immune checkpoint inhibitors. Furthermore, the nomogram constructed by integrating the Combined.NLR, histological type, PD−L1 (CPS) status, the number of prior therapy lines, and the presence of multi−organ metastasis provides a predictive model for ICI efficacy in this patient population. The findings offer a fresh perspective on assessing risks and developing tailored treatment strategies for the clinical management of metastatic or recurrent cervical cancer.

## Data Availability

The raw data supporting the conclusions of this article will be made available by the authors, without undue reservation.
