# Supplementary material for: Combined neutrophil-to-lymphocyte ratio and nomogram for predicting progression-free survival in recurrent/metastatic cervical cancer treated with immune checkpoint inhibitors
Source: Front Immunol. 2026 Apr 14;17:1811428. doi: 10.3389/fimmu.2026.1811428 (PMC13121155; doi:10.3389/fimmu.2026.1811428)
Supplement: Supplementary file 1 [file DataSheet1.docx]

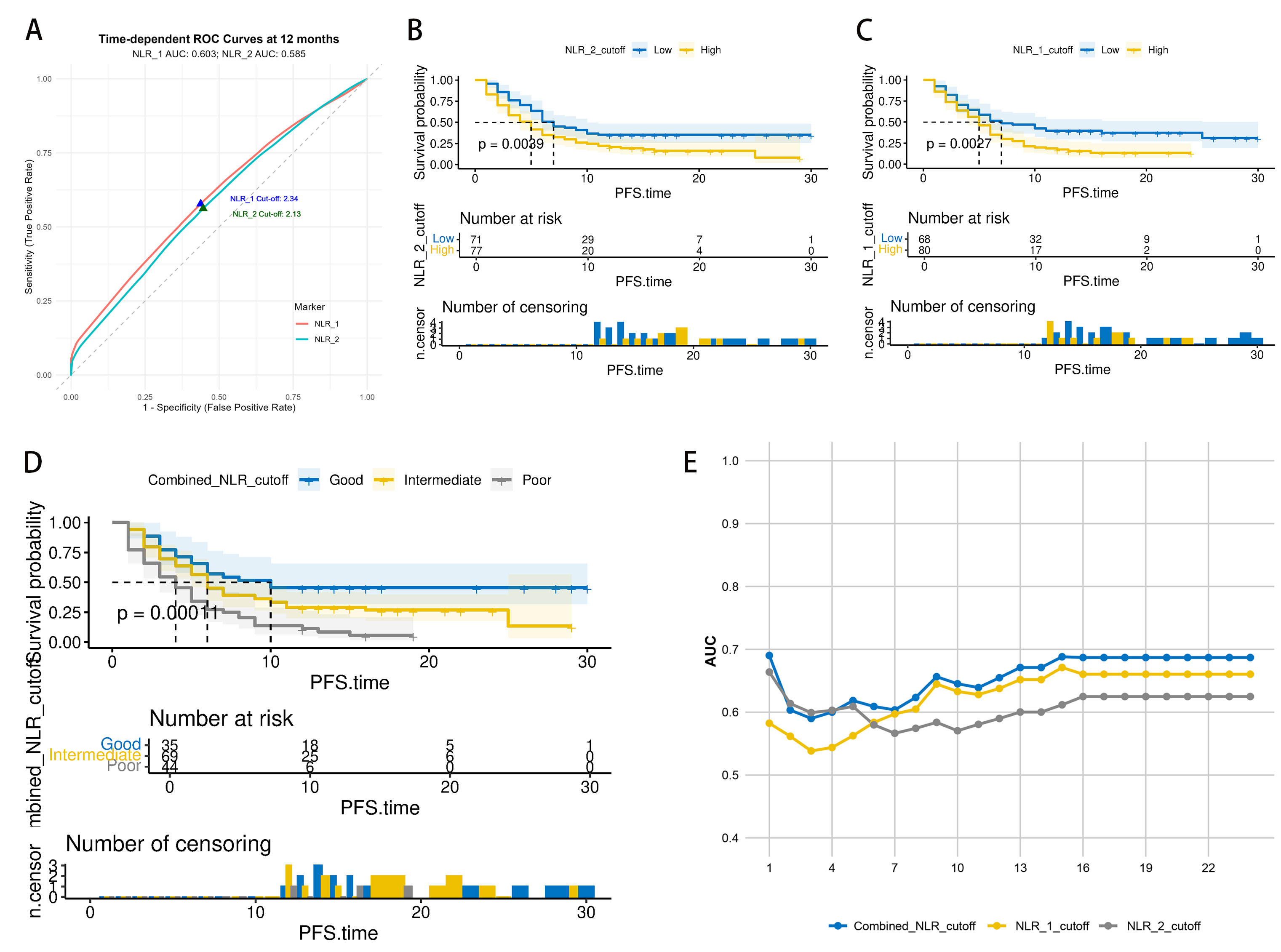


**Supplementary Figure 1. Time-dependent ROC curve analysis for determining optimal cut-off values of NLR_1, NLR_2, and Combined.NLR at 6 months.** (A) Time-dependent ROC curves and corresponding AUC values. (B–D) Kaplan–Meier curves stratified by ROC-derived cut-off values. (E) Dynamic AUC comparison across different time points. Although ROC-derived thresholds slightly improved predictive performance, median-based grouping was retained to enhance robustness and generalizability.


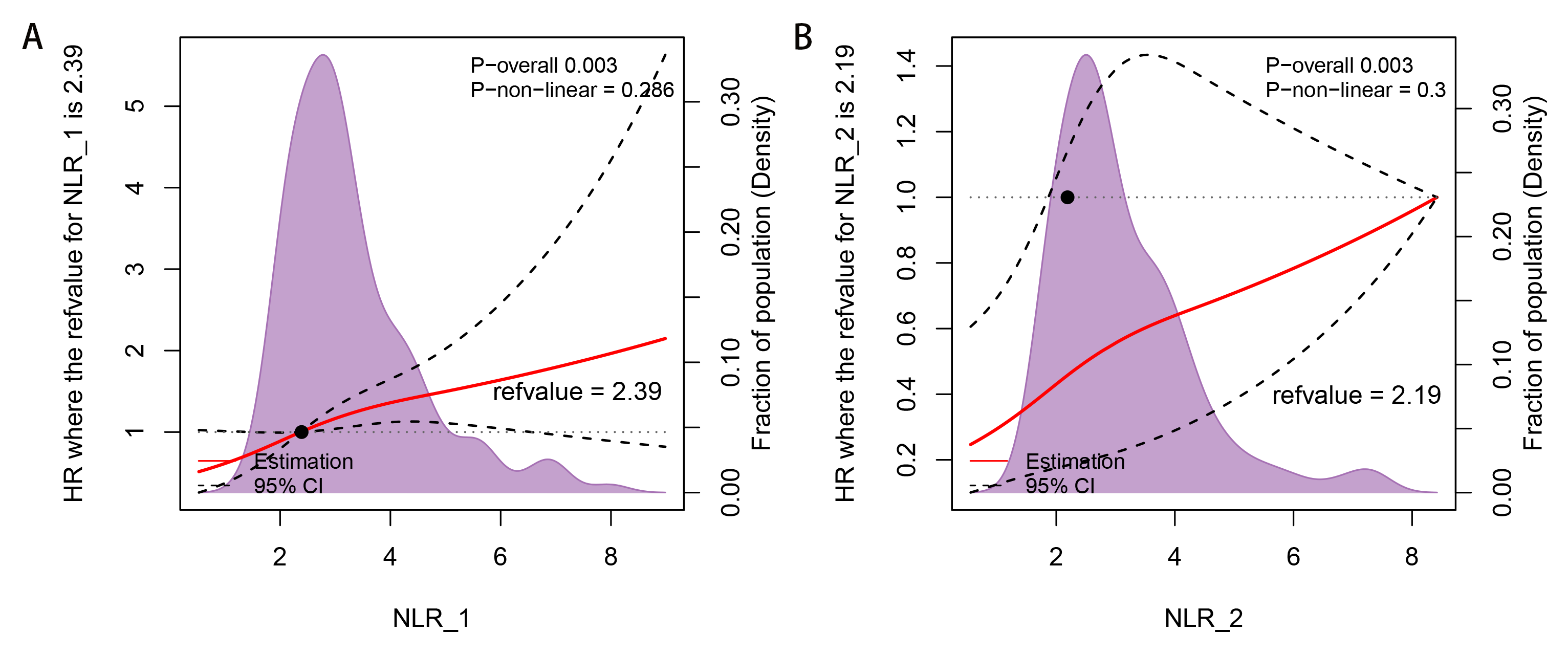


**Supplementary Figure 2. Restricted cubic spline analyses evaluating the association between (A) NLR_1 and (B) NLR_2 with progression-free survival.** The red lines represent hazard ratio estimates, and shaded areas indicate 95% confidence intervals. No significant nonlinearity was detected (P for non-linearity > 0.05), supporting linear modeling in Cox regression.
